# The World Café as a Tool for Evaluating the Level of Acceptance of SARS‐CoV‐2 Screening in School Settings, Puglia Region, Italy, 2023

**DOI:** 10.1111/hex.70137

**Published:** 2025-02-04

**Authors:** Valeria Gabellone, Fabiana Nuccetelli, Elisa Gabrielli, Leonardo Ascatigno, Pier Luigi Lopalco, Rosa Prato

**Affiliations:** ^1^ Department of Experimental Medicine University of Salento Lecce Italy; ^2^ Department of Prevention Local Health Unit Lecce Italy; ^3^ Department of Medical and Surgical Science University of Foggia Foggia Italy

**Keywords:** infectious disease, SARS‐CoV‐2, school, screening, students, World Café

## Abstract

**Background:**

The introduction of screening tests for Sars‐CoV‐2 has been an extraordinary prevention and control tool during the COVID‐19 pandemic. However, pandemic control interventions, including screening and vaccine mandates, induced refusal reactions in many people. To date, little information is available on the levels of acceptance of screening practices by the young population in school settings.

**Objective:**

The objective of this work is to survey students' attitudes, behaviours and emotions towards Covid‐19 screening tests by the means of a participatory research method, the World Café (WC).

**Materials and Methods:**

Between March and May 2023, three WC sessions were conducted in three high schools, with 70 students enrolled on a voluntary basis. As per standard procedure, a moderator was assigned at each table to facilitate dialogue and the WCs were recorded, transcribed and imported into ATLAS.ti software for qualitative analysis.

**Results:**

The analysis showed that the themes most reported during the WCs were those regarding the emotional domain, particularly feelings of distress, anxiety, fear, frustration, inadequacy and loneliness.

**Discussion:**

Although the themes ‘Emotions/thoughts’ appear to be the most prominent among students, also ‘Public health policies’ constitutes a predominant theme group. Finally, the theme ‘Communication’ sparked lively debate, being the fourth most discussed topic.

**Conclusions:**

The many insights from the WC analysis, when properly reframed, bring out useful elements for taking actions during prevention campaigns. Institutions and schools should focus on disseminating clear and targeted messages to help fight misinformation and distrust. Moreover, such evidence suggests that the World Café method proves to be useful and effective for exploring the emotional sphere of adolescents and analysing their thoughts, attitudes and knowledge.

**Patient or Public Contribution:**

The research team collaborated with the school personnel to set up the right setting for running the World Café sessions. The World Café method allowed the researchers to gather students' insights by the means of a participative process. Honest debate and active engagement of students provided the researchers with a list of statements very useful for informing health policy and recommendations.

## 1 Introduction

The public health emergency that accompanied the COVID‐19 pandemic highlighted the value of infectious disease prevention in public health. The collective ability to promote and protect this value has clearly become a decisive factor in societal safety, economic well‐being and development. The introduction of screening tests for Sars‐CoV‐2 has been an extraordinary prevention and infection control tool during the most difficult phases of the emergency [1]. However, health policy decisions implemented for pandemic control, including bans and mandates, inevitably induced negative reactions towards health authorities, such as feelings of distrust of vaccines and negative attitudes towards screening tests [2]. Yet, little information is available on how such issues were elaborated and affected the adolescent population in school settings. Despite the common agreement on the benefits of COVID‐19 screening as a tool to increase school attendance during the pandemic, adherence to school‐based screening programmes has often been suboptimal [3, 4]. The reasons behind the lack of compliance with such an important preventive measure may be complex. They can simply lie in fear for the discomfort the test may provoke or go deeply into personal beliefs and trust in health authorities [5].

To know attitudes, opinions and emotions of the population, during the last decades, there has been a growing interest in research techniques that exploit group interactions as a primary cognitive resource [6]. In this setting, social actors are not regarded as simple sources of information but as main actors of research capable of jointly elaborating a view of the phenomenon under investigation.

It is exactly within the theoretical context of Participatory Research (PR) that qualitative analysis becomes an effective tool for the transmission of information and, at the same time, an opportunity for the cultural growth of the participants. Qualitative research methods are used to explore and understand complex and contextual phenomena in the social sciences and also in the medical field. The aim of these analyses is to obtain an in‐depth understanding of the views, experiences and meanings attributed by the participants involved [7]. Based on these considerations, the World Café method (WC) was adopted and used in the present study as a qualitative research approach in school settings to carry out participatory work sessions based on participants' social interaction, a vehicle and resource for information transmission, role awareness and cultural growth.

Developed in America in the 1990s by J. Brown and D. Isaacs, WC is based on the concepts of collective intelligence and Bohmian Dialogue, that is, the realization that thinking is a collective process achievable only by the exchange of culture and communication and that intelligence emerges through common purposeful engagement among individuals in a group setting [8].

The scientific literature describes how the World Café method has been successfully applied to different contexts to promote collaboration and creation of projects aimed at improving living and working conditions. Within each discussion group, in fact, personal opinions are not only the result of individual reflections but come from collective discussion and confrontation with other participants [9]. This crucial aspect of the WC represents the first important aspect of the method and, for this reason, differs from traditional group interviews, where interaction takes place from time to time between the participants and the researcher.

As part of the project ‘Innovative systems for the early detection of COVID‐19 outbreaks in school settings in Italy’, work package ‘Determinants of Refusal of SARS‐CoV‐2 Screening in School Settings’, funded by the Italian Ministry of Health – CCM 2020 (National Centre for disease prevention and control), a conceptual model was developed to be used to set up standard tools for assessing individual barriers in adherence to preventive programmes such as screening tests for SARS‐CoV‐2. In developing the model, special emphasis was placed on factors that may be predominant in the school setting. The aim of this study was to analyse students' attitudes and to elicit their views on the topic based on their own experiences during the COVID‐19 pandemic.

## 2 Materials and Methods

Between March and May 2023, three World Café sessions were organized in three schools adhering to the Ministry of Health – CCM project in Puglia region, Italy, enrolling 70 high school students on a voluntary basis. Before each session, all participants signed an informed consent form containing a description of the method and details on the use of personal data.

A conceptual model based on five domains was developed for guiding the conversation sessions: swab taking, perception of disease risk, consequences of positivity, attitude towards authorities, information and communication.

The WC sessions carried out according to the guidelines of the World Café Community Foundation [10], involved 4 classes with a mean of 17.5 students per class distributed to 5 tables; each table had a moderator who was fixed at the established location whose task was asking questions, facilitating dialogue and noting emerging reflections. The discussion sessions included the use of some equipment, such as small tables and chairs distributed in the classroom (5–6 seats per table), placemats, sheets of paper and pencils to jot down thoughts, considerations and ideas. Each group of students rounded through all 5 tables at each turn change (every 20 min), thus providing their input to all discussion domains. The total duration of the sessions was, therefore, 1 h and 40 min (5 rounds, 20 min each), during which students addressed all the topics related to the domains of the conceptual model. The questions and topics addressed at the tables used as guide during the discussion sessions are summarized in Table 1.

**Table 1:** Discussion topics.

Domain	Question	Discussion points
**Swab execution**	*What can be done to facilitate screening for SARS‐CoV‐2 in the school setting?*	− Barriers and facilitators to acceptance of screening − Perception of the invasiveness of the test − Attitude towards salivary test versus nasopharyngeal swab
**Perception of disease risk**	*When will we be able to say that Covid‐19 is just another respiratory disease?*	− Severity of Covid‐19 (fatality, severity of illness, impact on absences from school or work) − Perception of current severity of Covid‐19 in frail individuals − Perception of the importance of diagnosis in setting specific therapy for Covid‐19
**Consequences of testing positive**	*If you have no symptoms or have common cold symptoms, does it make sense to swab?*	− Perception of the importance of diagnosis to limit the circulation of the virus and thus protect others − Consequences of knowing carrier status in terms of lost study time − Psychological consequences of positivity
**Personal attitude towards authorities**	*Could the government and health authorities have managed the pandemic better?*	− Assessment of the government's response to the pandemic − Government's role in health protection during the pandemic − Role of science in fighting the virus
**Information and communication**	*What information would you have liked to have received during the pandemic?*	− Usefulness and usability of the guidance given by the Ministry of Health − Usefulness and usability of the guidance given by LHU and GP − Usefulness and reliability of information about Covid‐19 found through social media and one's social network

All World Café sessions were recorded and transcribed in full, indicating the discussion domain and marking each participant's intervention with an identifying number from 1 to 6.

Data were analysed using inductive thematic analysis [11], which benefits from theoretical flexibility and the ability to categorize, organize and describe students' experiences through the identification of key themes and subthemes. The transcribed texts were imported into ATLAS.ti software, which, also takes advantage of artificial intelligence (AI), performed qualitative content analysis. In ATLAS. ti, categories can be renamed, deleted, grouped and merged by the researchers [12]. Two researchers independently examined the coding of the texts, refining it further after discussion and consensus. Codes and subcodes were grouped into themes, ensuring that these were consistent, clear and distinct.

The study was conducted after receiving ethics approval from the ethical committee of the University Hospital of Foggia number 62/CE/2023 on 29 March 2023. The study was conducted with the financial support of the Italian Ministry of Health – CCM 2020. The funder had no role in study design, data collection and interpretation, or decision to submit the work for publication.

## 3 Results

The arguments discussed by the 70 high school students included in the study, divided by the 5 thematic domains, listed in a matrix of favoring and hindering factors, are shown in Table 2.

**Table 2:** Arguments emerged from the discussions at each table of the World Cafè.

Domain	Facilitating factors	Hindering factors
**Swab execution**	− Minimal invasiveness (salivary vs. nasopharyngeal) − Ease of execution (self‐administration) − Free of cost	− Invasiveness − Cost − Difficult access
**Perception of disease risk**	− Peak period of illness − Confidence in therapy and vaccination − Presence of risk factors/contact with fragile people − Perception of severe or complicated Covid‐19 disease − Experience of acquaintances with severe COVID‐19 illness/sequelae/death − Perception of the effectiveness of the test as a public health measure − Possibility for frail subjects to receive therapies to reduce the risk of severe disease	− Low perception of viral circulation − Low perception of individual severity/real or presumed absence of risk factors − Recent asymptomatic/paucisymptomatic previous infection − Recent vaccination − Distrust in therapy − Lack of knowledge of therapeutic possibilities in case of positivity − Difficulty to perceive the effectiveness of the test as a public health measure
**Consequences of testing positive**	− Limited absence from school/work − Limited isolation/quarantine period − Awareness of the individual contribution to controlling the pandemic − Distancing from fragile people	− Unpaid absence from school/work − Stigma − Loss of social activities − Loss of school activities
**Personal attitude towards authority**	− Social involvement − Harmony with government choices − Trust in healthcare personnel/health authorities − Trust in scientific research	− Generic antigovernment attitude − Conspiracy/denialist attitude − Personal experience of mistrust in healthcare professionals
**Information and communication**	− Use of institutional sites, scientific journals, authoritative sources	− Use of noninstitutional sources − Social media

Transcripts of dialogues were categorized by ATLAS.ti into 641 codes and 107 themes, which were then combined to make increasingly generic theme groups. Finally, within the domains, the following 9 themes were identified and analysed:

1. Emotions and thoughts
2. Public health policies
3. Social behaviours
4. Media communication
5. Health perceptions
6. School management and impact
7. Perception of risk
8. Mental health
9. Personal experience

The details of the thematic map illustrating the relationships between the key themes and coded terms are provided in Table 3.

**Table 3:** Thematic analysis.

Themes	Codes	Exemplary statements
**Emotions/thoughts**	Fear, anxiety, anguish, confusion disappointment, scepticism, uncertainty trust, happiness, satisfaction, gratitude	Isolation from others, even having lunch alone, not feeling like you're with anyone, has created anxiety in each of us. (School A, Student 2)
**Public health policies**	Criticism of the government/school/health system agreement with government/school/health choices difficulty in accessing health services economy	The schools were poorly managed; the government did not provide the means for correct management of the distance learning. (School B, Student 2)
**Social behaviours**	Curiosity, conformism, altruism superficiality, ability to adapt, social influence, trust in science	The thing I liked about social media, for example TikTok or Instagram, is that an event as important in the pandemic was played down, there were some very ironic videos referring to COVID that showed another reality. There wasn't just widespread fear because a scared person watching those videos could perhaps have laughed and understood that the situation was serious and they had to be careful but panic was useless. (School B, Student 1)
**Media communication**	Fake news, reliability of sources, usability of communication, influence of social media	Communication during the pandemic was vast and social media reigned supreme… if you weren't careful and followed reliable sources, you came across dangerous fake news. (School C, Student 5)
**Perception of health**	Health concerns, susceptibility to diseases, psychophysical well‐being	In my opinion, avoiding getting swabbed allowed Covid to circulate more easily. Swabbing and screening are excellent strategies to prevent the disease and therefore to protect your health. (School C, Student 6)
**Management and school impact**	Poor school organization, problems with distance learning, learning difficulties, loneliness, teaching management	Even the return to school was poorly managed, half the class in distance and half in attendance, no one followed the lessons well, the teachers were not able to manage us. (School A, Student 1)
**Risk perception**	Risk minimization, compliance awareness, personal attitude health prevention	Today there is less pressure on hospitals, and this is already indicative of the more favourable course of the disease. Very few people need to be hospitalized, most are treated at home. (School B, Student 3)
**Mental health**	Well‐being, adaptation difficulties, discomfort, frustration, isolation, awareness	The waste of time of being positive, for example when you play sports and you are positive and you can't do it. (School B, Student 2)
**Personal experience**	Positive/negative experience, personal evaluation, level of health literacy, vaccination hesitancy	The swab was annoying, but it depends on the person who gives it to you, because it happened to me once when I was actually crying while they did it to me. While there were a few times when the person who did it to me had such a steady hand that I didn't even feel it. (School A, Student 3)

### 3.1 Swab Taking

This domain discussed the school prevention and screening measures taken during the high viral circulation phase of the pandemic.

Analysis of WC transcripts revealed discordant opinions with respect to each participant's experience. Some students expressed doubts and fears towards the invasiveness of the nasopharyngeal swab, although the stated concern was expressed as minimal:

*Seeing my classmates taking the swab in the classroom, I thought it was not a problem, because I could see them being relaxed about doing it…then, when it was my time, tears came out because it was really bothering me. In the end, though, it wasn't too invasive, just in that little bit of time.*

(School A, Student 1)

Looking deeper into the discomfort associated with the invasive practice of nasopharyngeal swabbing, some students reported such bad experiences that they no longer wanted to replicate the screening procedure or to reduce it to strictly necessary situations:

*I think that a swab should be less invasive, because in the end I, who had Covid, all along the duration of the disease, had the fear of these swabs, for this reason, for this kind of invasiveness.*

(School B, Student 3)

*I had a terrible experience with the nasal swab because the blood really came out. Yes, it hurt so much. Then I was afraid to do it later.*

(School C, Student 4)

The analysis of the transcripts also revealed several factors that facilitated the acceptance of the salivary swab in terms of minimal invasiveness, ease of performance and reduced cost:

*The salivary one is easier to do, quicker is less bothersome.*

(School C, Student 3)

*If the swab was free like the one done in school, it would be better and would be done more often. With some coughing someone wouldn't do it because of the price, instead if it wouldn't cost anything, it would be done more often.*

(School B, Student 2)

### 3.2 Perception of Disease Risk

In relation to perceived disease risk, students discussed the characteristics of the current phase of the pandemic, focusing on the perceived severity of COVID‐19. even in vulnerable individuals and the importance of diagnosis. In particular, within the theme group ‘social behaviours’, there was a tendency for students to minimize the severity of Covid‐19, which is generally considered a respiratory disease like so many others:

*I already consider Covid‐19 as a simple cold, as I myself, after recently contracting it, had mild symptoms that regressed in a few days.*

(School B, Student 1)

However, in reference to the most vulnerable individuals, students moved their subjective perception of disease risk towards a more careful and responsible attitude:

*The problem, in my opinion, at the moment is related more to fragile individuals who, if they contract the infection, may develop a more severe form of the disease. Young and healthy people, especially thanks to the introduction of vaccines, do not run this risk.*

(School B, Student 4)

### 3.3 Consequences of Positivity

The domain under examination addressed the consequences of testing positive in a screening test for Sars‐CoV‐2 and the importance of testing even asymptomatic or paucisymptomatic individuals, reflecting on the resulting positive in terms of loss of study time and the resulting psychological effects. It is well known that the pandemic has triggered feelings of concern in the general population related to the health conditions that any positivity would bring. Awareness of individual contribution to pandemic control and the importance of diagnosis to limit the circulation of the virus and protect others emerged among the students interviewed:

*It is a matter of public health, of protecting others. Knowing we are positive can help us and others protect their loved ones.*

(School C, Student 6)

Among the biggest barriers to adherence to Covid‐19screening, poor attitude to social distancing is noted, seen as a form of marginalization that can produce negative psychological effects in those who experience it:

*Although you stay inside the house, you cannot see even family members; you are completely alone. If you have a fever from another illness anyway your parents are there and help you; with Covid they are not.*

(School A, Student 3)

*Covid also caused loss of friendships, small groups were created and being positive and staying locked in the house caused us to lose friends.*

(School A, Student 2)

### 3.4 Personal Attitude Towards Authorities

Students' trust in health authorities was investigated to assess the degree to which they were attuned to government choices during the pandemic and their trust in science.

The qualitative analysis revealed students' partially critical attitude towards the government system that led the battle against COVID‐19 and disagreement with some of the policy choices implemented during the first phase of the pandemic, mainly in terms of social distancing and distance learning:

*Containment measures were too restrictive, school closures were poorly managed, the government did not provide the means for proper management of distance learning; the country was in a confusing and illogical management.*

(School C, Student 2)

*We were being bombarded with Prime Minister's Decrees and statistics; we were not leaving home and the infections were increasing, so obviously people were going out and there was no control.*

(School A, Student 2)

However, students expressed a general attitude of confidence in the health care system and scientific research, cornerstones in the fight against the virus:

*Science, however, was fast, and in the hospitals all the doctors and nurses did their best to protect the health of all of us.*

(School C, Student 5)

*Science helped defend against the virus with the vaccine.*

(School B, Student 2)

### 3.5 Information and Communication

The last domain explored participants' attitudes towards the different sources of information (institutional and noninstitutional) used during the pandemic, making personal opinions about the governmental management of communication in terms of the usefulness and usability of the guidance given by the Ministry of Health, local health units and the general practitioners.

Students reported a generalized sense of confusion with respect to the communication of the pandemic period, which emphasized, in many of them, prior perplexities:

*The information was handled well for those who could grasp it! The technical opinion of scientists helped but, on TV, everyone was saying their own and they had conflicting opinions, it was hard to understand what to do.*

(School C, Student 2)

There was a positive judgement on authoritative sources of information (institutional sites, ministerial guidelines, programmes and scientific journals) that fulfilled the need to disseminate clear and trustful messages:

*In my opinion, the bulletins issued periodically were useful because they gave people awareness of the trend curve of the virus and how it could and should be handled. I personally needed the Ministry of Health regulations to understand how to act; they were available on the Internet or printed in the shops, they were communicated about daily on talk shows and news programs.*

(School A, Student 3)

Finally, students' attitudes towards social media and unchecked sources of information appeared controversial. In fact, while social networks contributed to the spread of false and misleading messages, amplifying feelings of anxiety and distress in susceptible individuals, they helped to contrast the sense of isolation and deep loneliness generated by the pandemic:

*Social media helped a lot during the pandemic not to be isolated; for example, those who were alone in their room with Covid could network to know that they were not alone in that situation. If not used well, however, social media created panic and fake news.*

(School B, Student 4)

## 4 Discussion

Our study investigated the experiences, attitudes and beliefs of a group of high school students regarding the recent pandemic, with a focus on the impact of the screening methods for COVID‐19 offered for free in the schools as part of the Ministry of Health – CCM project ‘Determinants of Non‐Adherence to Screening for SARS‐CoV‐2 in School Settings’. The qualitative analysis revealed singular insights, and the World Café method enabled the creation of spontaneous and stimulating conversations, highlighting the young participants' keen critical sense and lively interest in the topics under discussion. The theme group ‘Emotions/thoughts’ turns out to be the most discussed by students within all 5 domains explored, confirming the centrality of the emotional sphere on adolescents' personal experience during the pandemic. Students reported some initial difficulties in adapting to the restrictions imposed by lockdown and distance learning, and this generated heterogeneous emotional responses with respect to isolation. Indeed, the emotional impact was multifaceted and diversified: some students reacted to the restrictions with proactive and supportive attitudes, while others experienced deleterious effects on their psyche, with anxiety, frustration, inadequacy and loneliness prevailing. In line with the results illustrated, recent scientific literature confirms that social isolation is a significant predictor of vulnerability to psychological distress in students [13], resulting in increased stress, anxiety and self‐reported depressive symptoms [14].

Many students have also expressed concern about the consequences of distance education on overall academic performance, claiming, in line with the literature, a decrease in the quality of achievement in terms of academic performance and interest in studying [15, 16].

‘Public Health Policies’ also constitutes a predominant theme in the dialogues among participants. Students' critical attitudes towards political governance evidently led to a significant reduction in trust towards institutions, which were accused of lack of transparency towards the population. Government management of the pandemic, restrictive measures, monitoring systems and ministerial decrees were contested in part by participants and often deemed excessive and negatively impacting individual stress levels. During the discussion sessions, preventive measures taken by schools during the high viral circulation phase were also examined, with largely favourable opinions from students regarding the desirability of being part of a free, noninvasive monitoring programme for SARS‐CoV‐2 in the school setting. Most participants firmly supported the great advantages of salivary swabbing, performed in the classroom, compared to nasopharyngeal swabbing, in terms of minimal invasiveness, convenience and speed of (self‐)execution, supporting its usefulness and effectiveness in the school setting. However, the aspects that emerged as major determinants of non‐adherence to screening for COVID‐19 are firmly related, once again, to the emotional sphere and personal experience of each participant. Continuing the analysis, the topic of communication sparked a lively debate, being the fourth most discussed topic in the various World Cafés. Pandemic information sources, often cited by students, embody the emblem of effective, correct and usable communication, as does the concept of misinformation, which has its roots in vaccine hesitancy. Indeed, students stated that confusing, often conflicting messages and misinformation about health risks can have a negative impact on personal attitudes, behaviours towards vaccines and adherence to screening campaigns. The scientific literature shows that a large proportion of young people get information about their health from social platforms [17, 18]; considering what has just been stated, the concern about the consequences to which the spread of misinformation among young people through social media can lead takes on unquestionable meaning. Indeed, this dangerous mechanism, if entrenched, would reinforce general distrust in institutional sources of communication, encouraging the establishment of attitudes of conspiracy, scepticism and vaccine hesitancy [19]. On the other hand, the position of students who stated that they use institutional sources as the exclusive channel from which to draw information regarding COVID‐19 is worth noting. This highlights a mature attitude and underscores how developing from a young age a good level of health literacy can guide towards conscious, knowledgeable choices that protect health even in situations of high psycho‐social impact, such as a pandemic. Finally, the opportunity for the participants to freely express their thoughts in the discussion sessions, by carrying personal experiences, underscored a sense of strong participation and emotional involvement towards all the issues addressed in the World Cafés.

This study has several strengths and limitations. The interest and participation in World Café by students show that it is an effective and appropriate approach to exploring students' experiences during the pandemic. However, data must be considered in terms of the context in which it was collected; the WC collected retrospective information on a small sample of participants coming from the same region of Southern Italy, although from different cities. Consequently, the present findings cannot be inferred to the entire adolescent population. Future research should therefore continue to implement in‐depth qualitative surveys of youth to document a variety of perspectives on their attitudes and beliefs. The interaction between the factors that have influenced adolescents' emotions and attitudes regarding the recent pandemic is complex and dynamic, and we do not claim to have been able to fully grasp it, as this would require a multifactorial approach. However, the results of this study could be used as a basis to further explore adherence to screening tests in the adolescent population during and after pandemics. In this respect, quantitative analysis may complement our findings by the means of robust statistically sound surveys.

## 5 Conclusions

The many insights gained from the WC analysis, if appropriately reframed, bring out useful elements for taking actions aimed at reducing young people's distrust and mistrust of vaccination and screening campaigns. Personal experience, whether positive or negative, was confirmed in the WC as a key determinant in guiding the health‐related choices of the students interviewed. Participants' partial criticism of government actions taken during the pandemic and little trust placed in institutions are fuelled by the lack of effective media communication, which left room for the spread of personal beliefs that turned out to be often wrong. Institutions and schools should therefore focus on disseminating clear and targeted messages to help fight misinformation and noncompliant behaviour. In the wake of the interest expressed by students regarding the preventive measures taken to respond to SARS‐CoV‐2 pandemic, it would be desirable to reintroduce similar initiatives aimed at educating and raising awareness among adolescents in the school setting. Pro‐social initiatives can, in fact, concretely strengthen the key factors of young people's adherence to screening campaigns [20].

In view of these considerations, a multifactorial approach and the cooperation of various stakeholders become necessary to discard generalized attitudes of scepticism and conspiracy and to facilitate the population's adherence to screening procedures. Finally, the results of the present study suggest that, in the context of qualitative analysis, the World Café method proves to be a useful and effective instrument for scrutinizing the emotional sphere of adolescents and analysing their thoughts, attitudes and knowledge, in the construction of a clear, spontaneous and unbiased dialogue in which everyone's opinion makes an exclusive and indispensable contribution.

## Author Contributions

**Valeria Gabellone:** investigation, formal analysis, data curation, writing–original draft, writing–review and editing, project administration, methodology. **Fabiana Nuccetelli:** methodology, data curation, investigation, formal analysis, writing–review and editing, project administration. **Elisa Gabrielli:** validation, project administration, writing–review and editing. **Leonardo Ascatigno:** investigation, validation, writing–review and editing. **Pier Luigi Lopalco:** conceptualization, methodology, writing–review and editing, validation, supervision. **Rosa Prato:** conceptualization, methodology, writing–review and editing, funding acquisition, validation, supervision.

## Conflicts of Interest

The authors declare no conflicts of interest.

## Data Availability

The data that support the findings of this study are available from the corresponding author upon reasonable request.
